# Biomimetic oriented nanofiber-reinforced conductive cardiac patch for enhanced cardiac repair after myocardial infarction

**DOI:** 10.1093/rb/rbag159

**Published:** 2026-07-23

**Authors:** Zheng Cao, Dong He, Linjie Zhong, Xiangyu Yu, Shuming Liu, Jiale Zhang, Xiaoling Fu, Yingjun Wang

**Affiliations:** School of Biomedical Sciences and Engineering, South China University of Technology, Guangzhou 511442, China; National Engineering Research Center for Tissue Restoration and Reconstruction and Innovation Center for Tissue Restoration and Reconstruction, Guangzhou 510006, China; Laboratory of Biomedical Engineering of Guangdong Province, South China University of Technology, Guangzhou 510006, China; School of Biomedical Sciences and Engineering, South China University of Technology, Guangzhou 511442, China; National Engineering Research Center for Tissue Restoration and Reconstruction and Innovation Center for Tissue Restoration and Reconstruction, Guangzhou 510006, China; Laboratory of Biomedical Engineering of Guangdong Province, South China University of Technology, Guangzhou 510006, China; School of Electronic and Information Engineering, South China University of Technology, Guangzhou 510006, China; School of Electronic and Information Engineering, South China University of Technology, Guangzhou 510006, China; School of Biomedical Sciences and Engineering, South China University of Technology, Guangzhou 511442, China; National Engineering Research Center for Tissue Restoration and Reconstruction and Innovation Center for Tissue Restoration and Reconstruction, Guangzhou 510006, China; Laboratory of Biomedical Engineering of Guangdong Province, South China University of Technology, Guangzhou 510006, China; School of Biomedical Sciences and Engineering, South China University of Technology, Guangzhou 511442, China; National Engineering Research Center for Tissue Restoration and Reconstruction and Innovation Center for Tissue Restoration and Reconstruction, Guangzhou 510006, China; Laboratory of Biomedical Engineering of Guangdong Province, South China University of Technology, Guangzhou 510006, China; School of Biomedical Sciences and Engineering, South China University of Technology, Guangzhou 511442, China; National Engineering Research Center for Tissue Restoration and Reconstruction and Innovation Center for Tissue Restoration and Reconstruction, Guangzhou 510006, China; Laboratory of Biomedical Engineering of Guangdong Province, South China University of Technology, Guangzhou 510006, China; School of Biomedical Sciences and Engineering, South China University of Technology, Guangzhou 511442, China; National Engineering Research Center for Tissue Restoration and Reconstruction and Innovation Center for Tissue Restoration and Reconstruction, Guangzhou 510006, China; Laboratory of Biomedical Engineering of Guangdong Province, South China University of Technology, Guangzhou 510006, China

**Keywords:** cardiac patch, conductive hydrogel, dopamine, PPy, myocardial infarction

## Abstract

The myocardium is a highly organized, multilayered anisotropic tissue in which the extracellular matrix (ECM) provides structural guidance for cardiomyocyte alignment, enabling synchronized contraction and efficient electrical conduction. Following myocardial infarction (MI), however, this intricate architecture and electrical integrity are severely disrupted, leading to impaired cardiac function and limited self-repair capacity. Consequently, engineering bio-inspired, multi-strata electroactive scaffolds capable of mimicking the essential architectural and physiological properties of natural heart tissue represents a vital strategy for successful cardiac repair. In this study, we constructed a biomimetic oriented nanofiber-reinforced conductive cardiac patch by integrating dopamine–polypyrrole (DA–PPy) with a GelMa hydrogel matrix. *In vitro*, the presence of multi-strata oriented nanofibers conferred directional electrical properties to the hydrogel while orchestrating the orderly arrangement of cardiomyocytes. This architecture ensured that cellular alignment followed the fiber axis within each layer, displaying a seamless transition across the stacked interfaces. In a rat MI model, the implanted cardiac patch exhibited remarkable therapeutic effects, including significant improvement of cardiac function, attenuation of ventricular wall thinning and fibrosis, reduced cardiomyocyte apoptosis and oxidative stress, and enhanced angiogenesis within the infarcted region. Furthermore, seeding cardiomyocytes onto the cardiac patch before implantation further amplified these reparative outcomes. Taken together, this work highlights the indispensable nature of re-establishing both the ECM’s structural integrity and its electrical properties to foster myocardial regrowth, while positioning our biomimetic conductive cardiac patch as a highly viable therapeutic modality for MI intervention.

## Graphical Abstract

**Graphical Abstract:**
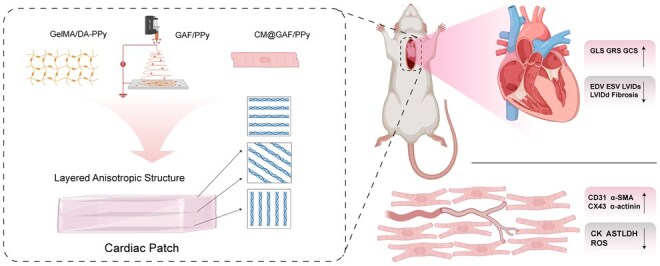

## Introduction

Globally, myocardial infarction (MI) stands as a primary cause of death [1], with an annual toll exceeding seven million [2]. Despite the widespread use of reperfusion strategies like thrombolysis [3], PCI [4] and CABG [5] to restore blood supply, these treatments cannot reverse the extensive loss of functional myocardium or the ensuing heart failure. Given the limitations of current interventions in restoring the damaged heart’s integrity, there is a pressing demand for advanced therapeutic paradigms designed to enhance myocardial healing and revitalize cardiac output post-MI.

Myocardial tissue, composed of extracellular matrix (ECM) and cardiomyocytes (CMs), is not only a dense, multilayered tissue [6, 7], but also a naturally electroactive tissue [8, 9]. ECM guides CMs to form anisotropic cardiac fibers which gradually shift in orientation from the endocardial to the epicardial layer. This architectural organization enables the heart to contract in a coordinated yet complex manner, while also contributing to efficient electrical conduction throughout the three-dimensional cardiac tissue. Following MI, this conduction network becomes disrupted, leading to irregular electrical signal propagation from non-infarcted region to the infarcted area. As a result, asynchronous contraction occurs in and around the damaged region of the myocardium [10]. Over time, persistent and excessive fibrosis replaces the damaged myocardium tissue with less elastic and poorly conductive scar tissue [11]. The presence of scar tissue further impedes the seamless flow of electrical signals across the boundary of the necrotic and healthy myocardium [12], which eventually triggers a significant decline in global cardiac performance during both contraction and relaxation phases. Thus, developing an engineered cardiac patch that replicates both the ECM architecture and electrical conductivity of healthy myocardium remains a significant goal in cardiac tissue engineering.

To address these challenges, researchers have explored innovative scaffold designs. A notable instance is the 3D hybrid system developed by Wu et al. [13], which features conductive nanofiber yarns with precise orientation encapsulated in a hydrogel shell. Such a platform effectively guides cell polarity and fosters the physiological maturation of CMs. Lin et al fabricated aligned nanofibrous patches seeded with CMs using electrospinning techniques. Their study demonstrated that CMs in anisotropic cardiac patches exhibited improved beating frequency, prevented electro-uncoupling and enhanced cardiac repair efficacy when implanted into a rat model of MI. However, many existing structural biomimetic approaches failed to replicate the multiscale architecture of the myocardium, particularly the controlled alignment and orientation shift between different layers [14, 15]. In terms of restoring the electrical connection within the infarcted myocardium, studies have demonstrated that incorporating conductive materials to re-establish electrical signaling and electromechanical coupling in damaged heart tissue is an effective strategy for enhancing cardiac function post-MI [16]. Significantly, the application of electroactive biomaterials as conductive interfaces has demonstrated the capacity to foster the maturation and functional integration of CMs, while simultaneously triggering the robust myogenic differentiation of stem cells. A case in point is the work by Li and colleagues, who fabricated a conductive scaffold by integrating a polypyrrole (PPy) and chitosan blend into gelfoam. This approach successfully enhanced electrical propagation speeds across the epicardial surface [17, 18]. Unfortunately, most current strategies focus either on mimicking the structural aspects of the myocardium or on restoring its electrical activity, but rarely address both simultaneously. This limitation often results in suboptimal outcomes for myocardial repair.

**Figure 1:**
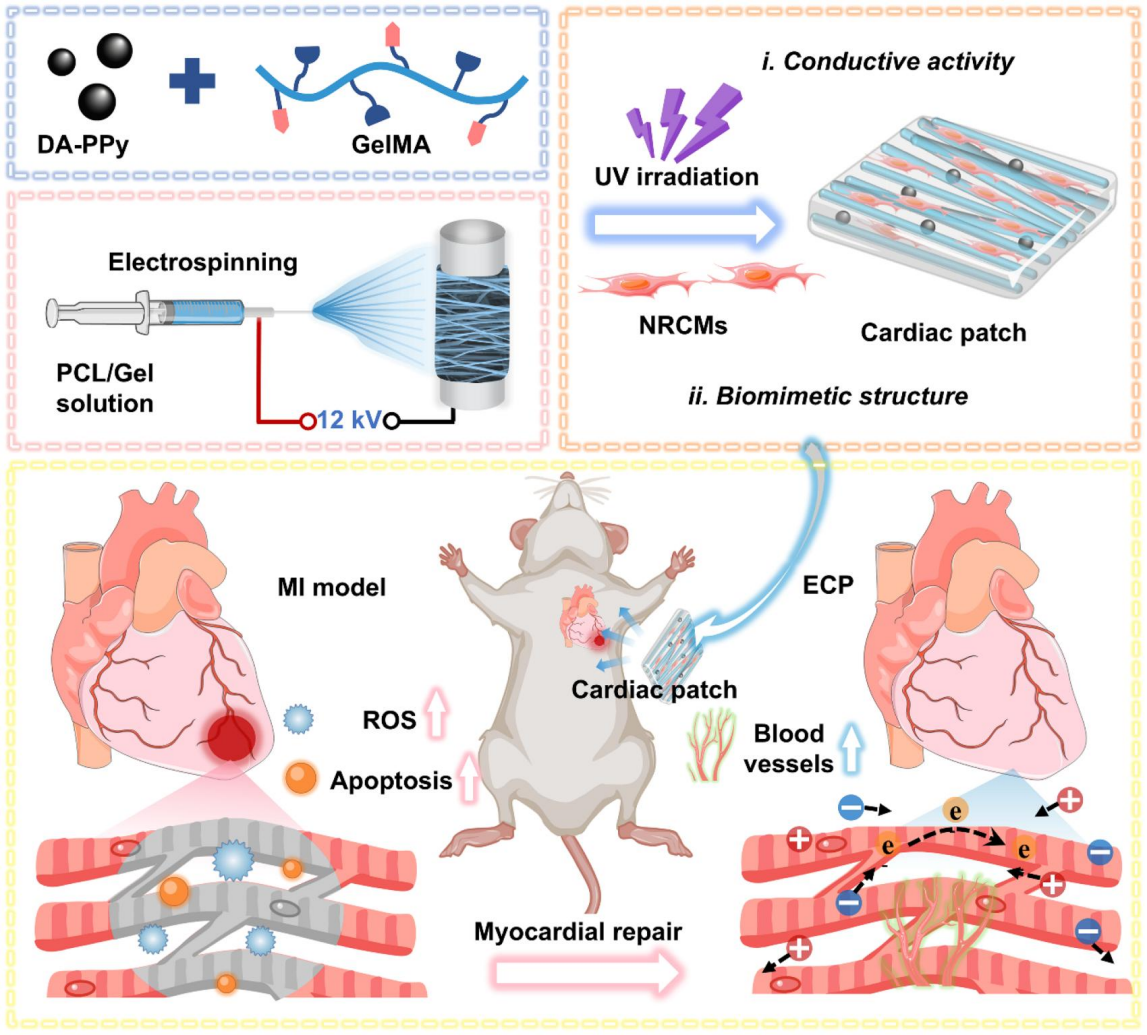
Preparation diagram of biomimetic oriented nanofiber-reinforced conductive hydrogel cardiac patch.

In the present study, a biomimetic patch was fabricated using a combination of aligned nanofibers and a conductive GAF/PPy hydrogel to provide both structural and electrical cues similar to the natural myocardium. By blending dopamine-modified polypyrrole (DA-PPy) with highly biocompatible GelMA, we aimed to bolster the electrical coupling of cardiac cells. The architectural complexity of the native tissue was further addressed by incorporating multiple plies of oriented electrospun fibers, ensuring a layered anisotropic organization within the composite. Additionally, primary CMs were incorporated as exogenous cells to augment the patch’s functionality. The regulatory effects of the patches on CMs were assessed through a series of *in vitro* experiments, and their therapeutic efficacy in repairing cardiac tissue post-MI was further evaluated through *in vivo* implantation in rats with MI (Figure 1).

## Materials and methods

### Materials and reagents

All essential reagents and primary materials utilized in the current research were procured from diverse commercial vendors. Polymer precursors, including Gelatin (porcine type A), polycaprolactone (PCL), pyrrole (Py) and FeCl_3_·6H_2_O, were acquired from Sigma-Aldrich (USA) and Macklin (China). Methacrylic anhydride (MA) and the photoinitiator lithium phenyl-2,4,6-trimethylbenzoylphosphinate (LAP) were sourced from Aladdin (China) and SinoBioPrint (China), respectively. For cell biological assays, Dulbecco's Modified Eagle Medium (DMEM), fetal bovine serum (FBS), penicillin/streptomycin (P/S) and trypsin/EDTA were provided by Gibco (USA), while the CCK-8 kit was from APE × BIO (China). Immunofluorescence staining was performed using phalloidin and primary antibodies against CX43, α-actinin, cardiac troponin T (cTnT) and CD31 (Abcam, UK), as well as α-SMA and WGA (ThermoFisher, USA). Furthermore, nuclear and functional dyes such as 4',6-diamidino-2-phenylindole (DAPI), Hoechst 33342 and Triton X-100 were supplied by Beyotime (China), with Calcein AM and Fluo-4 AM purchased from Yeasen (China).

### Fabrication and physicochemical evaluation of a conductive hydrogel reinforced with nanofibers based on GelMA and DA-PPy

#### Synthesis of GelMA and DA-PPy

The preparation of GelMA involved a methacrylation process. In brief, 5 g of gelatin was thoroughly solubilized in 50 mL of phosphate-buffered saline (PBS) at 60°C, followed by the controlled titration of MA at a rate of 0.5 mL/min. After allowing the reaction to proceed for 6 h, the solution was dialyzed against deionized water (MWCO: 12–14 kDa) for a duration of 7 days to ensure purification. The final GelMA product was harvested by collecting the supernatant after centrifugation at 2500 rpm, followed by a lyophilization process. An NMR spectrometer (Ascend 400, Bruker) was employed to verify the degree of substitution. To prepare modified DA-PPy, 0.12 mL of Py and varying amounts of dopamine hydrochloride (0–0.05 g) were mixed in 20 mL of 1 M HCl and chilled to 4°C. A solution of FeCl_3_·6H_2_O (1.35 g in 10 mL) was then introduced dropwise under constant stirring for 8 h. The resulting precipitates were isolated through centrifugation, rinsed five times with deionized water and freeze-dried for 24 h. The morphology and particle size were analyzed using SEM and DLS, respectively, while conductivity was assessed via a four-point probe.

#### Hydrogel synthesis

The conductive hydrogels (G10DP0.2, G10DP0.5) were formed by dispersing DA-PPy particles (0.2% or 0.5% w/v) into a 10% w/v GelMA solution containing 0.5% w/v LAP. After maintaining the precursor at 50°C for 30 min, 200 μL was cast into a PDMS mold and crosslinked under UV irradiation (405 nm, 25 mW/cm^2^, 180 s). To produce PCL scaffolds, an 8% (w/v) PCL/HFIP solution was electrospun following overnight stirring to ensure complete uniformity. The polymeric mixture was extruded through a 22-gauge stainless steel needle at a steady flow rate of 0.6 mL/h, driven by a 12-kV high-voltage field. Furthermore, the orientation of the fibers was strictly regulated by modulating the rotational speed of the aluminum foil-wrapped collector. Morphological evaluation of these electrospun meshes was conducted using a ZEISS Ultra 55 SEM, while fiber diameter distributions were analyzed based on these micrographs using ImageJ.

#### Fabrication and characterization of the composite patch

The nanofiber-reinforced conductive hydrogel was fabricated by infiltrating the PCL membrane (2 cm × 1.5 cm) with the GelMA/DA-PPy precursor solution. To evaluate the resulting composite, multiple characterization techniques were employed. The surface morphology was observed via scanning electron microscopy (SEM, ZEISS Ultra 55, Germany), while the optical properties were analyzed using a UV-vis spectrophotometer (UH5700, Japan). Rheological behavior and mechanical strength were assessed using a rotary rheometer (DHR-2, Waters) and a dynamic mechanical analysis (DMA) system (Q800, TA Instruments), respectively. Finally, the electrical conductivity was quantified via a four-point probe method using an RTS-8 source meter (Guangzhou Four-point Probe Technology, China). Tensile properties of the samples were evaluated using a universal testing machine (Instron 5967) equipped with a 100-N load cell. Rectangular specimens (20 mm × 15 mm × 0.5 mm) were used, with a gauge length of approximately 20 mm. The tests were conducted at a constant displacement rate of 1 mm/min. At least four specimens were tested for each group. Aligned PCL nanofibrous membranes (2 cm × 1.5 cm) were prepared via electrospinning (12 kV, 0.6 mL/h, 10 cm receiving distance) and treated with oxygen plasma for 3 min to improve hydrophilicity. Three layers of these membranes were stacked at preset angles (10°, 30°, 80°) to mimic the gradient orientation of native myocardium, and then placed into a PDMS mold. The GelMA/DA-PPy precursor solution (10% GelMA, 0.5% LAP, 0.2% DA-PPy) was gently injected to fully infiltrate the membrane layers and their interstices. Following a 180-s UV exposure at 405 nm (25 mW/cm^2^) to ensure network integration, the resulting 3D constructs were demolded and subjected to triple PBS washing.

### Cell culture

In terms of cell preparation, H9C2 rat cardiomyocytes (Procell Life Science & Technology, China) were cultured in a 5% CO_2_ incubator at 37°C using DMEM supplemented with 10% FBS and 1% P/S. For primary cardiomyocyte (CM) isolation, Sprague Dawley rats (within 24 h of birth) served as the source. Following a brief disinfection in 75% ethanol, neonatal hearts were harvested and transferred to chilled HBSS containing 10% P/S. The myocardial tissue was finely minced and underwent an overnight digestion process at 4°C with 0.25% trypsin/EDTA. To further dissociate the cells, the tissue was incubated with 0.05% type II collagenase (37°C, 190 rpm, 15 min), a cycle that was repeated until complete dissociation occurred. After neutralizing the suspension with high-glucose DMEM, the cells were passed through a 40-μm mesh and pelleted at 1000 rpm for 5 min. To enrich the CM population, differential adhesion was performed by pre-plating the cells for 90 min to deplete fibroblasts, with the purified non-adherent CMs subsequently harvested.

### Cell viability

To assess the cytocompatibility of hydrogels containing various DA-PPy concentrations (0, 0.2 and 0.5 mg/mL), both H9C2 cells and primary CMs were employed. For viability testing, cells were initially cultured for 24 h, followed by a 5-day incubation with the respective hydrogel treatments. Following PBS rinsing, the viability of the cells was assessed by incubating the samples with a Calcein-AM/PI live/dead assay kit for 30 min at 37°C under dark conditions. The resulting fluorescent signals were captured using a confocal laser scanning microscope (Zeiss LSM880), with the subsequent live cell percentage being determined through ImageJ software analysis.

In parallel, H9C2 proliferation was monitored using the CCK-8 assay. After introducing the hydrogels into the culture system, cells were maintained for 1, 4 and 7 days. The cell viability was assessed by substituting the supernatant with CCK-8 working solution at each scheduled interval. Subsequent to 2 h of incubation, the 450 nm optical density (OD) was measured via a microplate reader to quantify the metabolic activity.

### Characterization of CM calcium transients and spontaneous contraction behavior

To visualize intracellular calcium dynamics, CMs cultured on the hydrogels for three days were loaded with the Fluo-4 AM indicator. Following a 45-minute incubation period, the cells were transferred into a recording medium for functional assessment. The spatiotemporal calcium transients and corresponding contractile behavior were recorded using a live-cell imaging system at a temporal resolution of 10 frames per second. For data processing, the integrated fluorescence intensity (F) was normalized against the background or resting-state signal (F_0_) via ImageJ, allowing the subsequent analysis of the calcium flux profiles.

### Immunofluorescence

Following cultivation, cells underwent a sequence of fixation, permeabilization and blocking. Primary antibody incubation was performed at 4°C overnight using CX43 (15 μg/mL), α-actinin (1:100) and cTnT (0.5 μg/mL). Subsequently, cells were treated with fluorescently labeled secondary antibodies (1:1000) for 1 h at ambient temperature. To visualize the cytoskeletal organization and nuclei, phalloidin and DAPI were applied as counterstains. Confocal laser scanning microscopy (LSM880, Zeiss, Germany) was employed to capture the resulting fluorescence signals. To analyze angular differences between adjacent layers, cellular orientation was quantified based on co-staining of filamentous actin (F-actin, cytoskeleton) and DAPI (nucleus). Briefly, high-resolution fluorescence images were imported into ImageJ software. The major axis of individual nuclei and the alignment direction of nanofibers were manually defined using the ‘Straight’ tool, and the included angle between them was automatically calculated.

### MI model construction and treatment

With the authorization of the Animal Care and Use Committee at South China University of Technology (Approval No. 2024038), male Sprague Dawley (SD) rats, weighing 200–220 g (4 weeks of age), were maintained in a specific-pathogen-free (SPF) environment. To establish the MI model, animals were anesthetized via intraperitoneal injection of 3% sodium pentobarbital. Following a left thoracotomy to access the thoracic cavity, the left anterior descending (LAD) coronary artery was permanently ligated using 6-0 polypropylene sutures. Subsequently, the rats were randomly allocated into six therapeutic cohorts (*n* = 5–6 per group), including Sham (thoracotomy only), MI (infarction alone) and those receiving various patches: GAF (oriented fiber-reinforced GelMA), GRF/PPy (random fiber-reinforced conductive hydrogel), GAF/PPy (oriented fiber-reinforced conductive hydrogel) or CM@GAF/PPy (CM-laden GAF/PPy). Upon the respective cardiac patches were immediately transplanted onto the ischemic zone and secured to the epicardium using interrupted 7-0 non-absorbable polypropylene sutures around the border of the infarct area. The thoracic cavity was then closed, and the animals were monitored for postoperative recovery.

### Echocardiography and echocardiographic-based strain analysis

Four weeks post-transplantation, cardiac performance was evaluated using an ultra-high-frequency small animal ultrasound system (Vevo F2, VisualSonics, Canada). Key volumetric and functional parameters, including ejection fraction (EF) and fractional shortening (FS), were recorded. Additionally, structural dimensions such as Left ventricular internal diameter at end-systole (LVIDs), Left ventricular internal diameter at end-diastole (LVIDd), Left ventricular anterior wall thickness at end-diastole (LVAWd), Left ventricular anterior wall thickness at end-systole (LVAWs), End-systolic volume (ESV) and End-diastolic volume (EDV) were quantified to provide a comprehensive analysis of left ventricular remodeling.

Myocardial deformation dynamics, encompassing global and segmental strains (GLS, GRS, GCS) and their respective rates (GLSR, GRSR, GCSR), were determined via Vevo Strain Software. Longitudinal and radial strains were derived from parasternal long-axis views across six segments (from basal to apical regions). Meanwhile, circumferential and radial parameters were calculated from parasternal short-axis images covering the anterior free wall, lateral, posterior and septal regions. To ensure data reliability, all metrics represent the mean value of three successive cardiac cycles.

### Serum biochemical detection

On the 28th postoperative day, blood samples were harvested and processed via centrifugation to isolate serum for the assessment of myocardial injury markers. The concentrations of key diagnostic enzymes, specifically aspartate aminotransferase (AST), lactate dehydrogenase (LDH), creatine kinase (CK) and its MB isoenzyme (CK-MB), were subsequently quantified using a clinical biochemical analyzer (Hitachi, Japan).

### Histological assay

On the 28th postoperative day, harvested hearts were processed for either paraffin or frozen sectioning. For morphological analysis, specimens were fixed in 4% paraformaldehyde, embedded in paraffin and cut into 4-µm sections. hematoxylin and eosin (H&E) and Masson’s trichrome staining were performed to assess myocardial structure and fibrotic areas, with images acquired via a 3D Histech pathology scanner (Hungary). Infarct size and ventricular wall thickness were subsequently quantified using ImageJ.

To evaluate oxidative stress and apoptosis, 10-µm cryosections were incubated with 8-OHdG, IL-6 or TUNEL working solutions (30 min, 37°C) and counterstained with DAPI. For functional protein expression and vascularization, cryosections were fixed, permeabilized and blocked before overnight incubation at 4°C with primary antibodies: CX43 (1:60), α-actinin (1:100), cTnT (1:1500), CD31 (1:50) and α-SMA (1:500). Following treatment with fluorescent secondary antibodies (1:200, 1 h) and WGA/DAPI staining, fluorescence signals were captured using a Nikon Eclipse Ti2-E microscope. ImageJ was employed to determine the positive cell ratio, fluorescence intensity, blood vessel density and cardiomyocyte dimensions. The border zone was defined as the region within 500 µm of the infarct core–remote myocardium interface, characterized by mixed viable and fibrotic tissue. All representative immunofluorescence images and quantifications were obtained from the border zone.

### Statistical analysis

Statistical comparisons were conducted via GraphPad Prism 8, where all numerical results are reported as mean ± standard deviation (SD) from at least three separate biological trials. For datasets containing more than two groups, a one-way ANOVA was utilized. Differences were considered statistically significant when the *P* values was below the 0.05 threshold.

## Results and discussion

### Characterization of conductive GelMA hydrogel

**Figure 2:**
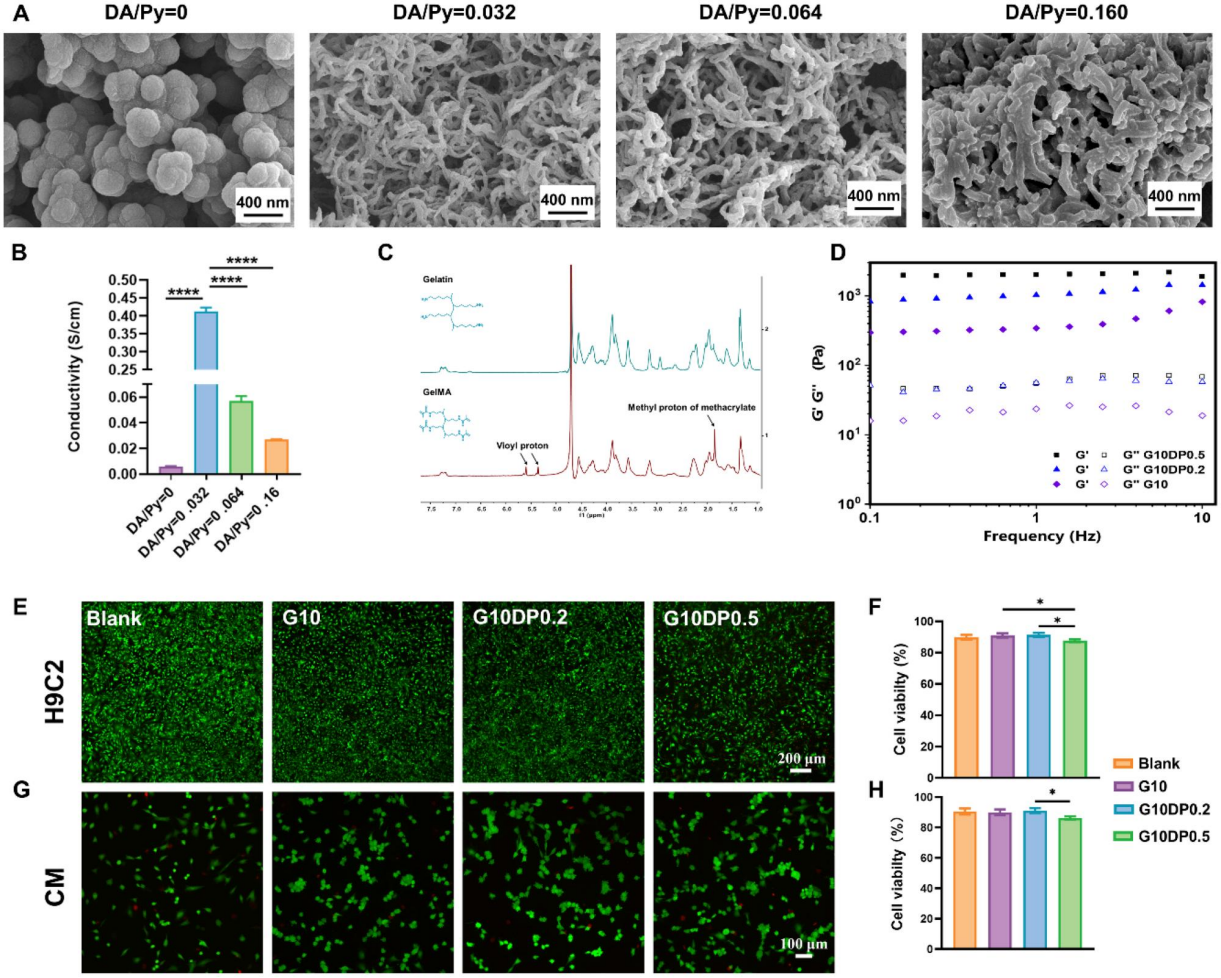
Synthesis and physicochemical profiling of the electroactive hydrogel. (**A**) Morphological features of DA-PPy electroactive particles across varying concentrations. (**B**) Electrical conductivity of the DA-PPy fraction as a function of particle concentration. (**C**) ^1^H NMR spectra of GelMA. (**D**) Rheological characterization of GelMA hydrogels with different DA-PPy contents. (**E**) Representative live/dead staining images of H9C2 cells cultured in a medium containing different hydrogel extract. (**F**) Viability of H9C2 cells after 3 days of exposure to different hydrogel extract. (**G**) Representative live/dead staining images of CMs cultured in a medium containing different hydrogel extract. (**H**) Viability of CMs after 3 days of exposure to different hydrogel extract. The experimental cohorts included DA/Py, Blank, G10, G10DP0.2 and G10DP0.5 (*n* = 4 per group). Results are expressed as mean ± SD. Statistical significance was determined by one-way ANOVA with Tukey’s *post-hoc* test. *P < 0.05, **P < 0.01, ***P < 0.001 and ****P < 0.0001.

SEM characterization revealed significant alterations in DA-PPy appearance upon dopamine modification. Specifically, the characteristic spherical geometry of PPy evolved into a fibrous, rod-shaped morphology following dopamine integration. Notably, elevating the DA/PPy ratio led to a concomitant increase in fiber thickness and abundance (Figure 2A). Notably, the incorporation of dopamine significantly enhanced the conductivity of PPy, among which the DA/PPy ratio of 0.032 showed the highest conductivity (Figure 2B). This improvement is likely due to DA promoting the assembly of PPy particles into fibers, while the inherent bonding properties of catechol group in DA facilitate more efficient charge transport. However, because catechol groups can attach to DA-PPy in various forms, the excess free catechol groups hinder electron transfer when the DA/Py ratio is high, leading to a reduction in conductivity [19]. Methacrylate anhydride was grafted onto the amino group of the gelatin side chains to form an amide bond, yielding double-bonded GelMA (Figure 2C). According to rheological characterization, the inclusion of DA-PPy conductive particles increased the modulus of the GelMA hydrogel as the DA-PPy content increased (Figure 2D). SEM characterization of the cross-sectional porous microstructure of GelMA/DA-PPy hydrogels at the tested DA-PPy concentrations (0, 0.2 and 0.5 mg/mL) has been supplemented (Supplementary Figure S1). The SEM images of the cross-sectional microtopography of G10 (10% GelMA), G10DP0.2 (10% GelMA + 0.2 mg/mL DA-PPy) and G10DP0.5 (10% GelMA + 0.5 mg/mL DA-PPy) hydrogels clearly show that the pure G10 hydrogel possesses a clear and interconnected porous network structure. After the incorporation of DA-PPy conductive particles, the microtopography of the hydrogel matrix changed significantly and DA-PPy conductive particles were clearly observed to be uniformly anchored on the porous network of the hydrogel. Quantitative analysis of the pore size revealed that the addition of DA-PPy particles reduced the pore size of the hydrogel network by approximately 2 ∼ 3 μm; yet, the GelMA/DA-PPy conductive hydrogels still maintained a distinct and interconnected porous structure, and no obvious agglomeration of DA-PPy particles was found in the hydrogel matrix. Subsequently, the cytocompatibility of the various conductive hydrogel formulations (G10, G10DP0.2 and G10DP0.5) was assessed by incubating H9C2 cells and CMs with hydrogel-derived extracts. As demonstrated by the live/dead staining assays (Figure 2E–H), the G10DP0.2 group exhibited the highest capacity for sustaining cell viability. These *in vitro* findings identified 0.2 mg/mL as the most suitable DA-PPy concentration for further study.

### Improvement of CMs function by the conductive GelMA/PPy hydrogel

**Figure 3:**
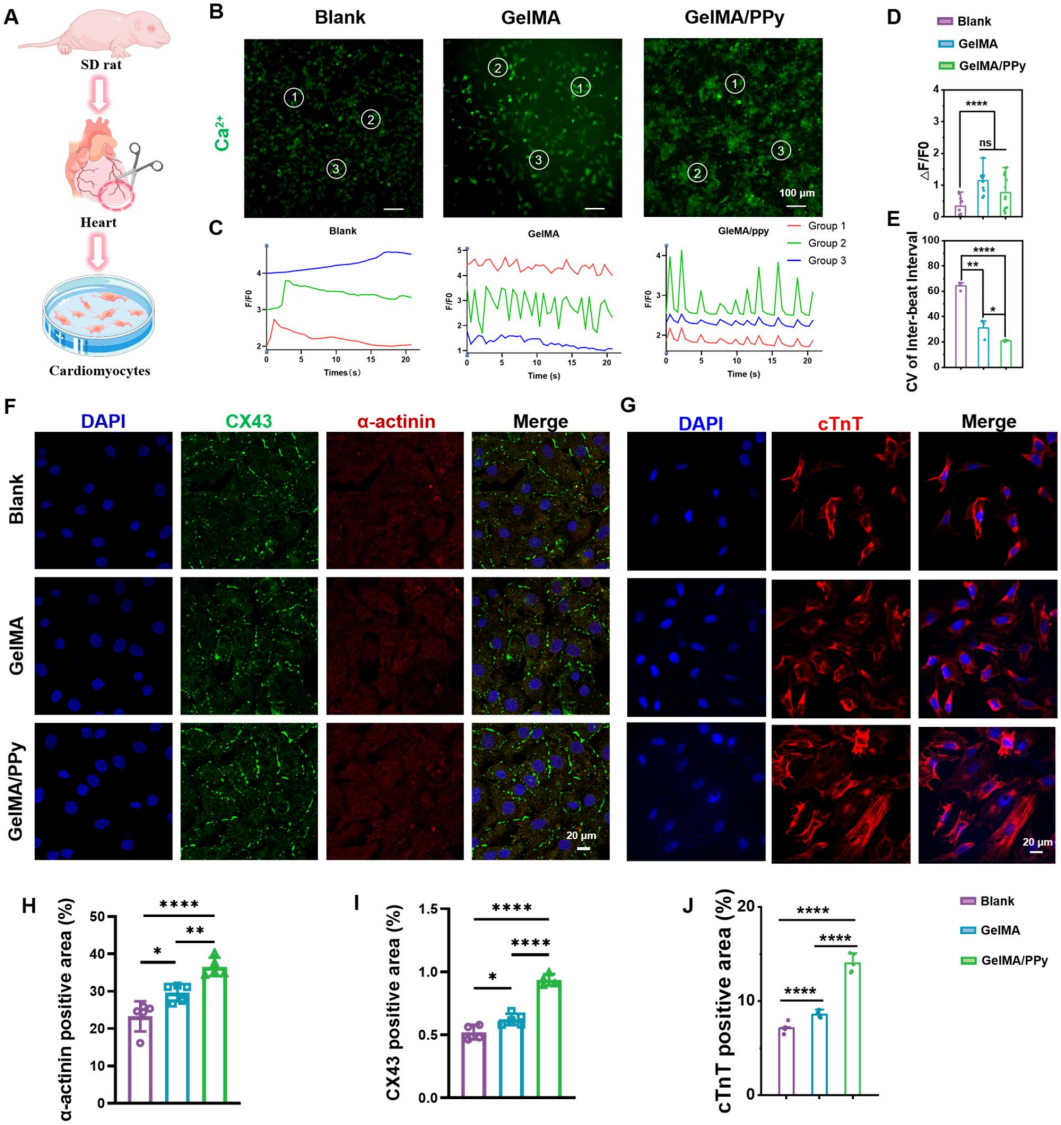
Effects of conductive GelMA/PPy hydrogel on the function of CMs. (**A**) Schematic illustration of rat primary cardiomyocyte extraction. (**B, C**) calcium transient signals and extracted frequency data within CMs cultured on different hydrogels on day 3. (**D**) ontraction amplitude and (**E**) frequency stability. (**F**) representative immunofluorescent images showing the expression of CX43 and α-actinin in CMs. Representative immunofluorescent images showing the expression of cTnT in CMs. (**H-J**) quantitative analysis of the positive area ratio for CX43, α-actinin and cTnT using ImageJ. Experimental cohorts included the Blank, GelMA and GelMA/ppy groups (*n* = 5 per group). Results are expressed as mean ± SD. Statistical significance was determined by one-way ANOVA with Tukey’s *post-hoc* test. *P < 0.05, **P < 0.01, ***P < 0.001 and ****P < 0.0001.

Calcium transient signals are critical to the contractility and conductivity of CMs, playing a pivotal role in regulating their overall function [20]. Thus, calcium currents in CMs cultured on different hydrogels were monitored by detecting fluorescence signals using the fluorescent calcium indicator, Fluo-4 AM. As illustrated in the imaging data (Figure 3B and Supplementary Movies S1–S3), synchronized Ca^2+^ spiking was clearly discernible within the GelMA/PPy hydrogel at the 3-day mark. This process was characterized by the development of parallel, rhythmic calcium waves propagating through various loci of the scaffold. In contrast, Ca^2+^ puffs in CMs from the blank group lacked both linearity and rhythmicity, displaying varying frequencies across different regions (Figure 3C). Furthermore, we addressed the functional aspect by performing further quantitative analysis to evaluate contraction amplitude and frequency stability. As shown in Figure 3D and E, the experimental group exhibited significantly higher calcium transient amplitude (reflecting contraction strength) and significantly lower beat-to-beat interval variability (reflecting more stable contraction frequency) compared to controls. These functional parameters provide direct evidence of enhanced cardiomyocyte maturation.

To evaluate the regulatory effects of the GelMA/PPy hydrogel on cardiomyocyte functionality, we assessed the expression of key cardiac markers, including α-actinin, connexin-43 (CX43) and cardiac troponin T (cTnT), across different hydrogel substrates. α-Actinin, a hallmark of cardiomyocyte maturation, is closely associated with contractile function [21], while CX43, predominantly localized at intercellular junctions, plays a critical role in electrical impulse propagation and electromechanical coupling in the myocardium [22]. cTnT, a cardiomyocyte-specific protein, is also indicative of contractile machinery integrity. Immunofluorescence analysis at day 3 (Figure 3D–G) revealed that the GelMA/PPy matrix significantly upregulated the expression of CX43, α-actinin and cTnT compared with the GelMA and blank groups. The above results indicate that the addition of PPy significantly enhanced the maturity of CMs and improved the electrical coupling between cells.

### Characterization of biomimetic oriented nanofiber-reinforced conductive cardiac patch (GAF/PPy)

**Figure 4:**
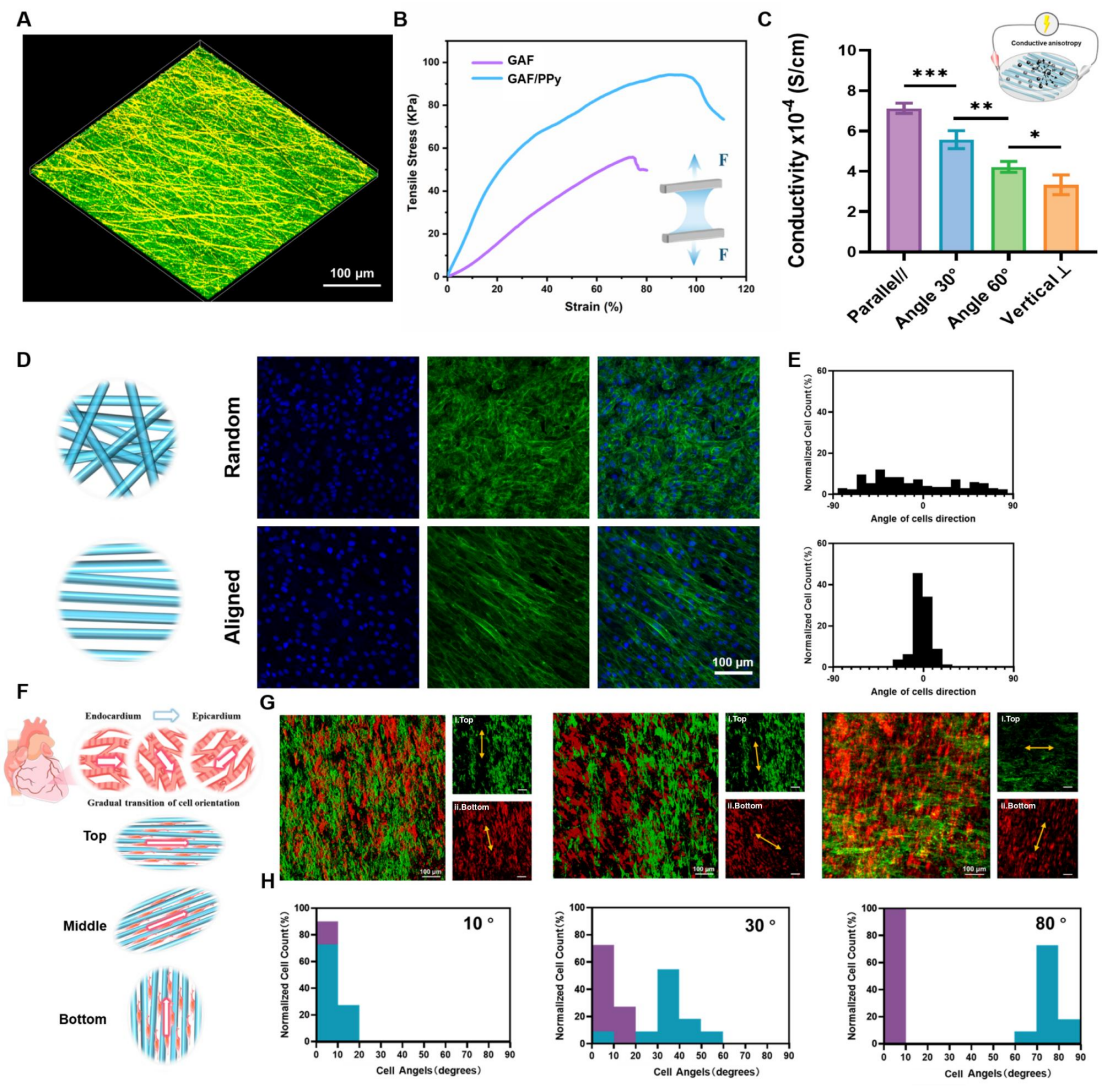
Characterization of biomimetic oriented nanofiber-reinforced conductive cardiac patch. (**A**) 3D confocal imaging of single-layer cardiac patch. (**B**) Tensile property testing of different cardiac patches, GAF, biomimetic oriented nanofiber-reinforced cardiac patch; GAF/PPy, biomimetic oriented nanofiber-reinforced conductive cardiac patch. (**C**) Conductive anisotropy of the cardiac patch. (**D**) Representative fluorescence image of H9C2 cells cultured on nanofibers with different orientations for 3 days. (**E**) Statistical analysis of cell orientation angles. (**F**) Diagrammatic representation of the myocardium depicting the incremental shift in cardiomyocyte alignment from the endocardium toward the epicardium, paired with a model of multilayered scaffolds featuring a stepwise rotation of fiber orientations. (**G**) Representative fluorescence images of H9C2 cells on different fiber layers with different orientations. (**H**) Statistical analysis of angle differences between adjacent layers. Experimental groups consisted of parallel (*∥*), 30°, 60° and vertical (⊥) alignments (*n* = 4 per group). Results are expressed as mean ± SD. Statistical significance was determined by one-way ANOVA with Tukey’s *post-hoc* test. *P < 0.05, **P < 0.01, ***P < 0.001 and ****P < 0.0001.

Firstly, we prepared two types of nanofibers through electrospinning, namely random nanofibers and oriented nanofibers. The SEM images are shown in Supplementary Figure S2. Next, the nanofiber-reinforced conductive cardiac patch was prepared by incorporating nanofibers and conductive GelMA/PPy hydrogel. The 3D confocal imaging illustrated the aligned nanofiber structure within 3D hydrogel matrix (Figure 4A). Given the cyclic mechanical demands of the beating heart, cardiac patches must possess appropriate mechanical properties to withstand and support dynamic contraction. The oriented nanofiber-reinforced cardiac patch (GAF) exhibited relatively limited tensile strength, whereas incorporation of rigid DA-PPy into the aligned nanofiber-reinforced conductive patch (GAF/PPy) markedly enhanced its mechanical performance (Figure 4B). Mechanical compatibility with native myocardium is essential for long-term functionality, as substantial mismatches may induce interfacial stress and impair contractile coordination. The GAF/PPy patch exhibited a tensile strength of 93.21 kPa, which falls within the range of native myocardial tissue reported for pig (79.699 kPa), fresh bovine (83.984 kPa), sheep (111.514 kPa) and preserved bovine heart (114.278 kPa) [21–23], indicating a reasonably matched mechanical profile. Electrical conductivity measurements revealed a clear directional dependence: as the current direction shifted from parallel to perpendicular relative to the fiber alignment, the conductivity decreased accordingly (Figure 4C). This behavior suggests that the incorporation of aligned nanofibers modulates the spatial distribution of conductive DA-PPy particles, thereby imparting anisotropic electrical properties to the composite patch. These results confirm the successful fabrication of an aligned nanofiber-reinforced conductive cardiac patch at the single-layer level. Native myocardium exhibits anisotropic electrical conductivity, with reported values ranging from 0.16 S/m (murine) to 0.57 S/m (human), and a longitudinal-to-transverse conductivity ratio of approximately 3.2 in canine myocardium [21, 22]. In comparison, the GAF/PPy patch displayed anisotropic conductivity (7.13 ± 0.24 × 1 0^−4^ S/cm parallel vs. 3.34 ± 0.42 × 10^−4^ S/cm perpendicular; anisotropy ratio ≈ 2.14), partially recapitulating the directional conduction characteristics of native cardiac tissue and supporting intercellular electrical communication. Future refinements—optimizing DA-PPy dispersion, adjusting fiber density or adding conductive additives—could better match native electrical properties.

Consistent with previous studies, fibers with different orientations significantly influenced cell morphology (Figure 4D). Specifically, CMs cultured on aligned fibers exhibited excellent alignment and elongation, with approximately 80% of the cells oriented between −9° and 9° (Figure 4E). These results indicated that the prepared aligned fibers could be used as ideal substrates to guide CMs to form natural myocardium.

In native cardiac tissue, CMs are uniformly oriented within each layer, with a gradual transition of alignment between layers (Figure 4F). Given the necessity of autonomous regulation over cellular alignment within complex tissues, we developed a hierarchical biomimetic scaffold featuring three distinct layers of oriented fibers (10°, 30°, 80°). This design aimed to evaluate the patch’s efficacy in orchestrating 3D cellular orientation and elongation (Figure 4G). Following 3 days of culture, fluorescence imaging confirmed that H9C2 cells had successfully infiltrated all layers, strictly adhering to the pre-defined fiber directions of each stratum. This layered alignment was further substantiated by quantitative polar plots (Figure 4H), highlighting the scaffold’s superior capacity to replicate the intricate structural anisotropy found in native myocardium.

### Enhancement of cardiac function by biomimetic oriented fiber-reinforced conductive cardiac patches following MI

**Figure 5:**
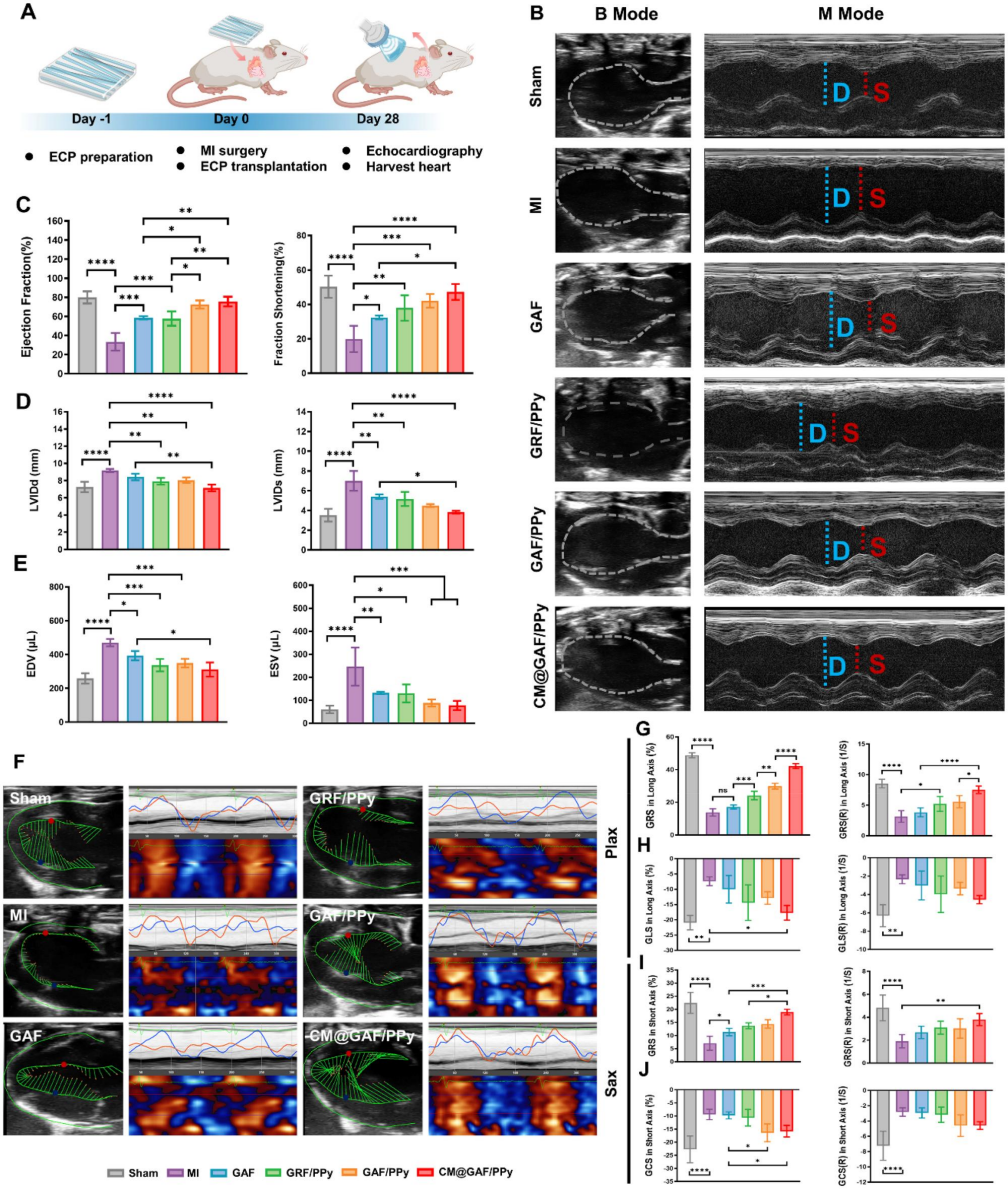
Therapeutic effects of different cardiac patches in rats with MI. (**A**) Schematic illustrating the study design of the animal experiment. Cardiac function was assessed 28 days post-MI. (**B**) Typical B-mode and M-mode echocardiographic images illustrating the anatomical configuration and contractile dynamics of the left ventricle. Evaluation of (**C**) LVEF and LVFS, (**D**) LVIDd and LVIDs and (**E**) EDV and ESV using Vevo Lab software; (**F**) Representative Vevo strain and motion heatmap images. (**G-J**) Analysis of global radial strain (GRS) and global radial strain rate (GRSR) in the parasternal long axis (Plax) (**G**), global longitudinal strain (GLS) and global longitudinal strain rate (GLSR) in Plax (**H**), GRS and GRSR in the parasternal short axis (Sax) (**I**) and global circumferential strain (GCS) and global circumferential strain rate (GCSR) in Sax (**J**) using Vevo Lab software. Experimental cohorts comprised the Sham, MI, GAF, GRF/PPy and GAF/PPy groups (*n* = 5 per group), along with the CM@GAF/PPy group (*n* = 6). Results are expressed as mean ± SD. Statistical significance was determined by one-way ANOVA with Tukey’s *post-hoc* test. *P < 0.05, **P < 0.01, ***P < 0.001 and ****P < 0.0001.

To evaluate the therapeutic efficacy of the cardiac patch *in vivo*, we induced MI in a rat model and administered four distinct treatments to separate groups (Figure 5A). The defective regeneration capacity of CMs leads to irreversible loss of tight-joint cytoarchitecture in the infarct area [21], but cell-implantation strategies offer a promising paradigm for cardiac repair after MI. CMs were loaded onto GAF/PPy (hereafter referred to as CM@GAF/PPy) as an exogenous cell supplement to the infarct sites. Cardiac function was assessed using echocardiography on day 28 post-MI. In the MI group, classical signs of MI were observed, including stiff left ventricular (LV) anterior wall activity and LV dilation. In contrast, the different cardiac patches-treated groups displayed improved cardiac activities, with the CM@GAF/PPy and GAF/PPy groups showing the most pronounced LV anterior wall contraction (Figure 5B). Quantitative echocardiographic evaluation highlighted severe cardiac impairment in the untreated MI cohort, evidenced by markedly diminished EF and FS, coupled with pathological ventricular expansion (increased EDV and ESV). Conversely, intervention with various cardiac patches effectively ameliorated these hemodynamic deficits. Notably, the CM@GAF/PPy group demonstrated the most robust functional restoration (Figure 5C), with substantial reductions in ventricular dimensions (LVIDs, LVIDd) and volumes (EDV, ESV) (Figure 5D and E). The acellular GAF/PPy group showed comparable therapeutic efficacy across most parameters, except for ESV. These functional gains in the GAF/PPy and CM@GAF/PPy cohorts are likely driven by the synergistic effects of DA-PPy-facilitated electrical conduction and the biomimetic mechanical guidance of aligned nanofibers, while exogenous CM supplementation in the latter further accelerated tissue repair. To evaluate contractile synchronicity, a speckle-tracking algorithm was utilized to visualize myocardial wall kinetics. Heatmap analysis (Figure 5F) revealed superior motion coordination in patch-treated hearts, with the CM@GAF/PPy and GAF/PPy groups exhibiting the most uniform displacement patterns. This mechanical synchrony was further validated by point-specific motion curves and multiaxial strain analysis. Statistical quantification (Figure 5G–J) confirmed that the absolute values of longitudinal, radial and circumferential strain and strain rates were significantly elevated in the GRF/PPy, GAF/PPy and CM@GAF/PPy groups relative to the MI group.

The serum biochemical testing results show that the levels of AST, LDH, CK and its CK-MB were all reduced after treatment with various cardiac patches, indicating decreased release of myocardial enzymes from damaged myocardial tissue and repaired myocardial cell necrosis (Supplementary Figure S4). Among them, the GAF/PPy and CM@GAF/PPy treatment groups exhibited a stronger inhibitory effect on the release of CK and LDH, demonstrating that both cell-laden and acellular cardiac patches effectively reduced the degree of myocardial injury.

**Figure 6:**
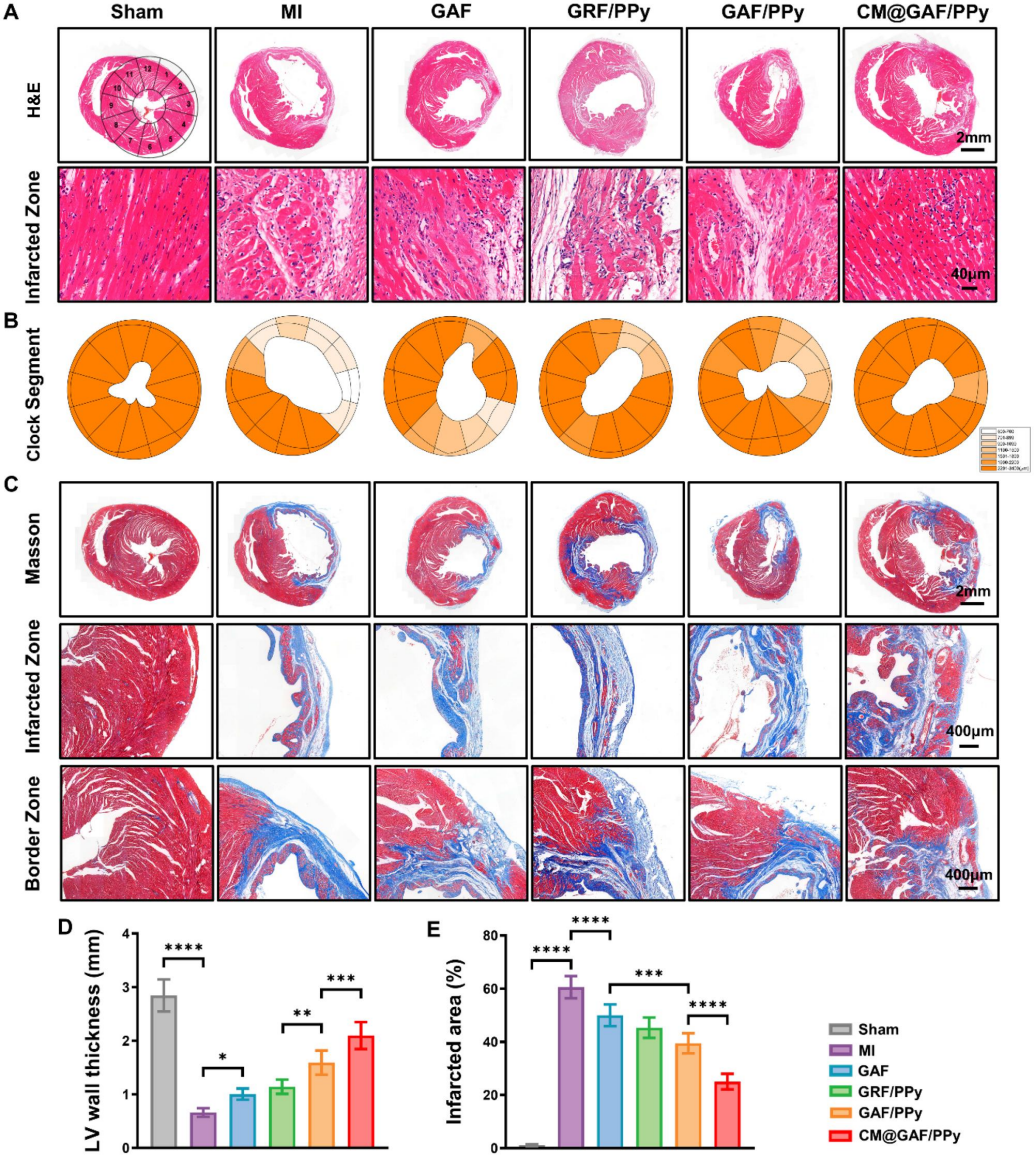
Histopathological evaluation of rat hearts at 28 days following MI induction. (**A**) Typical H&E-stained sections of the entire heart and magnified regions within the infarct area. (**B**) Assessment of LV wall thickness across 12 discrete segments. (**C**) Representative Masson’s trichrome staining showing the gross heart structure and high-magnification views of the infarct and peri-infarct regions. (**D–E**) Statistical quantification of the LV wall thickness and the total infarct size. *n* = 5 for Sham, MI, GAF, GRF/PPy and GAF/PPy groups; *n* = 6 for CM@GAF/PPy. Results are expressed as mean ± SD. Statistical significance was determined by one-way ANOVA with Tukey’s *post-hoc* test. *P < 0.05, **P < 0.01, ***P < 0.001 and ****P < 0.0001.

Histological assessment using H&E staining further confirmed the echocardiographic observations regarding ventricular wall dimensions (Figure 6A). Pronounced LV remodeling, characterized by chamber dilation and wall thinning, was evident in the MI group. Conversely, the application of cardiac patches significantly mitigated wall thinning within the infarcted area; the CM@GAF/PPy group showed the greatest improvement in wall thickness, outperforming the GAF/PPy group (Figure 6D). The clock segment map, which partitioned the LV into 12 segments and color-coded thickness levels, corroborated these findings (Figure 6B). Masson’s trichrome staining showed that the GAF/PPy group significantly reduced fibrotic area. However, treatment with CM@GAF/PPy resulted in the least fibrosis in both the infarct and boundary areas among all treatment groups (Figure 6C and 6E). These results indicated that the CM@GAF/PPy effectively restored the heart’s physiological structure, alleviated ventricular wall thinning, inhibited fibrosis scar formation and improved myocardial retention. Collectively, these findings underscore the capacity of the engineered patches to reverse pathological LV remodeling and restore synchronized systolic function, with the CM@GAF/PPy construct yielding the most potent therapeutic outcomes.

The profound *in vivo* therapeutic efficacy of the GAF/PPy patch is rooted in the potent synergy between topographical alignment and anisotropic conductivity, which collectively replicates the complex 3D electromechanical syncytium of native myocardium. In the healthy heart, CMs form anisotropic fibers that undergo a gradual orientational shift across the ventricular wall, dictating both the principal axis of mechanical contraction and the preferential pathway for electrical propagation. In our design, the aligned PCL nanofibers not only provide direct mechanical guidance for cardiomyocyte elongation and cellular alignment, but also sterically influence the distribution of DA-PPy within the GelMA matrix, yielding a marked conductive anisotropy. When these individual anisotropic layers are stacked with sequential rotational shifts to mimic the physiological endocardial-to-epicardial transition, they construct a biomimetic microenvironment that offers simultaneous structural and electrical directional cues. This structural-electrical synergy is crucial: the topographical confinement forces the cells into a physiological morphology, while the anisotropic conductive network establishes robust, directional electrophysiological coupling between these aligned cells. This is evidenced by the prominent upregulation of CX43 and α-actinin, and the generation of highly synchronous, rhythmic calcium transients. Upon implantation, this engineered construct functions as an active electromechanical bridge, translating these *in vitro* maturation cues into organized, synchronous myocardial contraction and preventing the formation of electrically inert, structurally chaotic fibrotic scar tissue.

The cardiac patch is designed as a biodegradable construct. *In vivo* experiments showed that 28 days after transplantation into the MI area of rats, the patch remained tightly attached to the cardiac surface with an intact morphology, without obvious detachment or structural disintegration (Supplementary Figure S5). Its biodegradability is enabled by the inherent degradation pathways of constituent materials: GelMA undergoes enzymatic hydrolysis, PCL degrades via ester bond hydrolysis, and DA-PPy (low dosage: 0.2% w/v) is gradually cleared as the matrix degrades.

### Promoted myocardium survival and angiogenesis by biomimetic oriented fiber-reinforced conductive cardiac patches following MI

**Figure 7:**
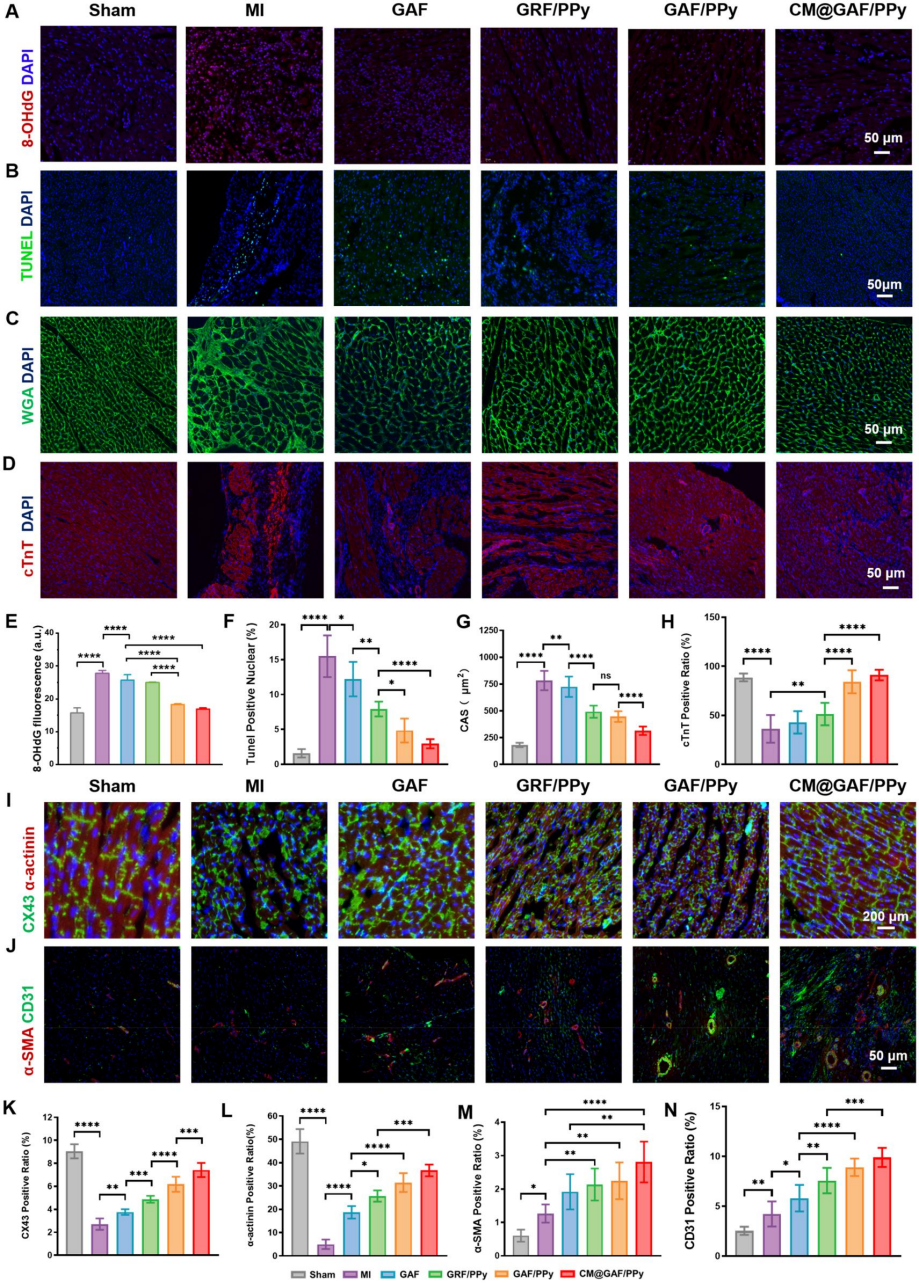
Fluorescent staining of rat cardiac tissue to explore the potential therapeutic mechanism of different cardiac patches. All representative immunofluorescence images and quantifications were obtained from the border zone. (**A**) Representative fluorescent images showing the ROS levels of MI rats via 8-OHdG staining. (**B**) Representative fluorescent images showing the cell apoptosis of MI rats via TUNEL staining. (**C**) Representative fluorescent images showing the cardiomyocyte profiles of MI rats via WGA staining. (**D**) Representative fluorescent images showing the expression of cTnT. (**E**) Average fluorescence intensity of 8-OHdG analyzed using ImageJ. (**F**) The TUNEL positive nucleus ratio analyzed using ImageJ. (**G**) Myocyte size measured using ImageJ. (**H**) the positive ratio of cTnT analyzed using ImageJ. (**I**) Representative fluorescent images showing the expression of CX43 and α-actinin. (**J**) Representative fluorescent images showing the expression of α-SMA and CD31 via immunofluorescence staining. (**K-N**) The positive ratios analyzed using ImageJ of CX43, α-actinin, α-SMA and CD31. Experimental cohorts comprised the Sham, MI, GAF, GRF/PPy and GAF/PPy groups (*n* = 5 per group), along with the CM@GAF/PPy group (*n* = 6). Results are expressed as mean ± SD. Statistical significance was determined by one-way ANOVA with Tukey’s *post-hoc* test. *P < 0.05, **P < 0.01, ***P < 0.001 and ****P < 0.0001.

Following MI, excessive oxidative stress creates a detrimental microenvironment characterized by elevated reactive oxygen species (ROS), which subsequently induces oxidative DNA damage and triggers cardiomyocyte (CM) apoptosis [22]. To comprehensively evaluate this cascade, 8-OHdG and TUNEL staining were performed to assess ROS accumulation, oxidative DNA lesions and programmed cell death within the infarct region (Figure 7A, B, E and F). At 28 days post-MI, 8-OHdG staining revealed pronounced oxidative DNA damage in infarcted myocardium, which was substantially alleviated in all patch-treated groups, with the CM@GAF/PPy group demonstrating the most pronounced protective effect (Figure 7A and E). Correspondingly, the elevated CM apoptosis observed in the MI group was markedly attenuated following patch implantation, as indicated by a reduced density of TUNEL-positive nuclei (Figure 7B and F). Collectively, these findings indicate that the patches effectively mitigate the oxidative stress–DNA damage–apoptosis cascade, thereby improving the hostile post-infarction microenvironment. Notably, the superior efficacy of the CM@GAF/PPy patch can be attributed to the synergistic interplay of multiple factors: the intrinsic free radical-scavenging capability of dopamine within the PPy-based matrix attenuates ROS-mediated injury; the conductive and anisotropic microenvironment facilitates electrical signaling and cellular organization; and the delivered CMs further contribute through paracrine secretion and direct participation in tissue repair. This multifaceted synergy enables more effective restoration of myocardial homeostasis.

Furthermore, the impact of the treatments on compensatory hypertrophy was assessed via WGA staining of CMs in the distal peri-infarct regions (Figure 7C). The CM@GAF/PPy group demonstrated a superior capacity to restrain pathological CM enlargement compared to both MI and other scaffold-treated groups (Figure 7G). Immunofluorescence evaluation of the infarct area (Figure 7D and H) revealed that the GAF/PPy and CM@GAF/PPy patches not only enhanced cTnT expression but also improved myocardial preservation, leading to a highly organized tissue architecture. The CM@GAF/PPy treatment also significantly upregulated CX43 and α-actinin (Figure 7I), indicative of restored electrical coupling. A comparable trend in the GAF/PPy group suggests that the synergy between biomimetic topographic guidance and conductive hydrogel properties facilitates inter-cellular impulse propagation, effectively mimicking native myocardial signaling (Figure 7K and L).

The restoration of vascular networks is fundamental to the structural and functional recovery of the infarcted myocardium. To assess the pro-angiogenic potential of various cardiac patches *in vivo*, the expression of angiogenesis-specific markers, CD31 and α-SMA, was visualized using immunofluorescence (Figure 7J). While negligible angiogenic activity was detected in the healthy myocardium, significant neovascularization was observed across all treatment groups. Notably, the fluorescence intensities of CD31 and α-SMA were considerably higher in the CM@GAF/PPy and GAF/PPy groups than in the MI group, demonstrating their superior capacity for stimulating blood vessel formation within the ischemic zone (Figure 7M and N).

To assess tissue inflammation after cardiac patch implantation, we detected IL-6 expression by immunostaining. IL-6 levels in the infarcted myocardium were significantly elevated in the MI group relative to the Sham group, demonstrating severe inflammatory injury induced by MI. In contrast, IL-6 expression in the infarcted region was notably decreased in all cardiac patch treatment groups, with the greatest reduction seen in the CM@GAF/PPy group. This prominent anti-inflammatory effect is largely ascribed to the anti-inflammatory properties endowed by the introduction of exogenous CMs. Detailed experimental data, statistical analyses and representative images for IL-6 detection are provided in Supplementary Figure S6.

Collectively, these findings demonstrated that the CM@GAF/PPy and GAF/PPy patches effectively promoted cardiomyocyte survival and functions, contributing to the restoration of cardiac function following injury.

The *in vivo* therapeutic efficacy of the GAF/PPy patch was tightly linked to its *in vitro* regulation of cell behaviors, forming a ‘structural biomimicry-cellular functional maturation-*in vivo* tissue repair’ regulatory chain. *In vitro*, the patch’s multilayer oriented nanofibers effectively guided the alignment of H9C2 cells and neonatal rat cardiomyocytes (NRCMs), with ∼80% of cells oriented within −9°∼9° (Figure 4E), which recapitulates the anisotropic structural feature of native myocardium. This oriented cell arrangement, coupled with the patch’s anisotropic conductivity (7.13 ± 0.24 × 10^−4^ S/cm parallel to fibers), promoted synchronous calcium transients (Figure 3B and C) and upregulated the expression of gap junction protein CX43 and contractile protein α-actinin (Figure 3D–F), enhancing intercellular electro-mechanical coupling. Such *in vitro* cellular functional maturation laid the structural and functional foundation for *in vivo* repair: after transplantation, the oriented fiber-guided cellular alignment within the patch facilitated the reconstruction of anisotropic myocardial tissue at the infarct site, reducing disorganized fibrosis (Masson staining, Figure 6C–E) and restoring the ordered electrical conduction pathway. Consequently, the *in vivo* myocardial strain synchronization (Figure 5F–J) and left ventricular function (EF, FS improvement, Figure 5C) were significantly enhanced, as the oriented cell network mimics native myocardium’s directional contraction and electrical propagation. Collectively, the patch’s *in vitro* modulation of cell orientation, electro-mechanical coupling and vascular endothelial cell activity directly contributes to its *in vivo* effects of mitigating ventricular remodeling, restoring conduction synchrony and improving cardiac function, which is consistent with the notion that biomimetic structural cues and conductive properties synergistically promote myocardial repair [8, 22].

Despite the promising therapeutic efficacy of the GAF/PPy patch, several inherent limitations merit discussion. Firstly, functional assessments (e.g. calcium transient analysis) were not performed for the aligned PCL/GelMA/PPy composite group. While the conductive GelMA/PPy group alone demonstrated functional maturation benefits, and the composite group showed enhanced structural maturation, direct evidence of functional synergy between alignment and conductivity remains to be established in future studies. Secondly, regarding the physical application, the patch lacks intrinsic tissue adhesion, necessitating surgical suturing with 7-0 polypropylene threads. This procedure increases the risk of epicardial injury and bleeding, potentially compromising therapeutic outcomes [23]. The absence of *in situ* bonding motifs, such as catechol-containing moieties, further restricts its potential for minimally invasive delivery. Moreover, the technical complexity of accurately implanting the multilayered anisotropic structure on the infarcted myocardium remains a significant challenge, which may prevent the full realization of its biomimetic potential during surgical procedures. Thirdly, the reliance on NRCMs presents a major barrier to clinical translation. Beyond the ethical concerns and lack of scalability associated with the sacrifice of neonatal animals, NRCMs are xenogeneic cells prone to immunogenicity and rejection in human applications [24]. Furthermore, their inherent electrophysiological divergence from human physiology and their structural immaturity characterized by a fetal-like phenotype and metabolic reliance on glycolysis may limit their long-term contractile performance and functional translatability. In parallel, the risk of pro-arrhythmia cannot be overlooked, as incomplete electromechanical coupling between exogenous cells and host tissue may create ectopic pacemakers [11, 25]. Moreover, the hostile post-infarction microenvironment, marked by hypoxia, inflammation and fibrotic scarring, poses a significant challenge to cell survival and seamless integration with the healthy myocardium. Future research should prioritize the utilization of clinically applicable cell sources, such as human induced pluripotent stem cell-derived cardiomyocytes (hiPSC-CMs), while developing advanced pre-vascularization strategies to enhance the patch’s integration and functional maturation [26]. Ultimately, advancing strategies for functional maturation and pre-vascularization will be pivotal to optimizing the therapeutic efficacy of iPSC-loaded conductive patches.

## Conclusion

In summary, we developed a biomimetic oriented nanofiber-reinforced conductive cardiac patch (GAF/PPy). This cardiac patch integrated the advantages of electrical conductivity provided by DA-PPy modified GelMA hydrogel, the biomimetic oriented structure and reinforced mechanical property provided by aligned nanofibers. This patch guided the arrangement and elongation of cardiomyocytes and promoted cardiomyocyte maturation *in vitro*. The i*n vivo* animal studies further validated that GAF/PPy provided significant cardio protection, as evidenced by marked recovery of cardiac function, a substantial improvement in cardiomyocyte function and improved angiogenesis in the infarcted region. Notably, the GAF/PPy patch loaded with cardiomyocytes (CM@GAF/PPy) exhibited superior therapeutic effects. This work presents a new approach for the design and construction of patches for cardiac tissue engineering.

## Supplementary Material

rbag159_Supplementary_Data
