# Supplementary material for: Biomimetic oriented nanofiber-reinforced conductive cardiac patch for enhanced cardiac repair after myocardial infarction
Source: Regen Biomater. 2026 Jul 23;13:rbag159. doi: 10.1093/rb/rbag159 (PMC13484876; doi:10.1093/rb/rbag159)
Supplement: Supplementary file 1 [file rbag159_supplementary_data.zip › Supporting Information (final upload).docx]

**Supporting Information**

Biomimetic oriented nanofiber-reinforced conductive cardiac patch for enhanced cardiac repair after myocardial infarction

Zheng Cao^1, 2, 3,†^, Dong He^1, 2, 3,†^, Linjie Zhong^4^, Xiangyu Yu^4^, Shuming Liu^1, 2, 3^, Jiale Zhang^1, 2, 3, *^, Xiaoling Fu^1, 2, 3, *^, Yingjun Wang^1, 2, 3^

^1.^ School of Biomedical Sciences and Engineering, South China University of Technology, Guangzhou International Campus, Guangzhou 511442, P. R. China

^2.^ National Engineering Research Center for Tissue Restoration and Reconstruction and Innovation Center for Tissue Restoration and Reconstruction, Guangzhou 510006, P. R. China.

^3.^ Laboratory of Biomedical Engineering of Guangdong Province, South China University of Technology, Guangzhou 510006, P. R. China.

^4.^ School of Electronic and Information Engineering, South China University of Technology, Guangzhou 510006, P. R. China.

^*.^ Correspondence address. School of Biomedical Sciences and Engineering, South China University of Technology, Guangzhou International Campus, Guangzhou 511442, P. R. China. Email: msxlfu@scut.edu.cn.

^†.^ These authors contributed equally to this work.

Movie S1. The calcium transient signals in CMs on the blank hydrogel.

Movie S2. The calcium transient signals in CMs on the GelMA hydrogel.

Movie S3. The calcium transient signals in CMs on the GelMA/PPy hydrogel.

Figure S1 (A) Micromorphology of hydrogel. (B) Hydrogel pore size statistics.

Figure S2 Random nanofibers and oriented nanofibers.

Figure S3 Statistics of serum important myocardial enzymes after 28 days of cardiac patch treatment. (A) Serum CK levels; (B) Serum CK-MB levels; (C) Serum AST levels; (D) Serum LDH levels. (Experimental cohorts comprised the Sham, MI, GAF, GRF/PPy, and GAF/PPy groups (n = 5 per group), along with the CM@GAF/PPy group (n = 6). Results are expressed as mean ± standard deviation (SD). Statistical significance is denoted as follows: *p < 0.05, **p < 0.01, ***p < 0.001, and ****p < 0.0001.)

Figure S4 The combination of heart patch and heart of rats in each group. Twenty-eight days post-implantation, harvested hearts showed that while the MI group exhibited extensive ventricular blanching and severe injury, the Sham group remained intact. In contrast, the cardiac patches maintained robust epicardial adhesion and structural integrity. Post-patch removal, the underlying myocardium showed significant morphological recovery compared to the MI group, confirming that the scaffolds provide sustained mechanical coupling and prolonged therapeutic effects.

Figure S5 IL-6 Fluorescent staining of rat cardiac tissue to explore the potential therapeutic mechanism of different cardiac patches. (A) Representative fluorescent images showing the IL-6 levels of MI rats; (B) The quantitative analysis using ImageJ. (Experimental cohorts comprised the Sham, MI, GAF, GRF/PPy, and GAF/PPy groups (n = 5 per group), along with the CM@GAF/PPy group (n = 6). Results are expressed as mean ± standard deviation (SD). Statistical significance is denoted as follows: *p < 0.05, **p < 0.01, ***p < 0.001, and ****p < 0.0001.)
